# Impact of Salivary Gland Hypertrophy Virus Infection on the Mating Success of Male *Glossina pallidipes*: Consequences for the Sterile Insect Technique

**DOI:** 10.1371/journal.pone.0042188

**Published:** 2012-08-13

**Authors:** Gratian N. Mutika, Carmen Marin, Andrew G. Parker, Drion G. Boucias, Marc J. B. Vreysen, Adly M. M. Abd-Alla

**Affiliations:** 1 Insect Pest Control Laboratory, Joint FAO/IAEA Programme of Nuclear Techniques in Food and Agriculture, Vienna, Austria; 2 Entomology and Nematology Department, University of Florida, Gainesville, Florida, United States of America; Kansas State University, United States of America

## Abstract

Many species of tsetse flies are infected by a virus (GpSGHV) that causes salivary gland hypertrophy (SGH). Female *Glossina pallidipes* (Austen) with SGH symptoms (SGH**+**) have reduced fecundity and SGH**+** male *G. pallidipes* are unable to inseminate female flies. Consequently, *G. pallidipes* laboratory colonies with a high prevalence of SGH have been difficult to maintain and have collapsed on several occasions. To assess the potential impact of the release of SGH**+** sterile male *G. pallidipes* on the efficacy of an integrated control programme with a sterile insect technique (SIT) component, we examined the mating efficiency and behaviour of male *G. pallidipes* in field cages in relation to SGH prevalence. The results showed in a field cage setting a significantly reduced mating frequency of 19% for a male *G. pallidipes* population with a high prevalence of SGH (83%) compared to 38% for a male population with a low prevalence of SGH (7%). Premating period and mating duration did not vary significantly with SGH status. A high percentage (>80%) of females that had mated with SGH**+** males had empty spermathecae. The remating frequency of female *G. pallidipes* was very low irrespective of the SGH status of the males in the first mating. These results indicate that a high prevalence of SGH+ in *G. pallidipes* not only affects colony stability and performance but, in view of their reduced mating propensity and competitiveness, releasing SGH+ sterile male *G. pallidipes* will reduce the efficiency of a sterile male release programme.

## Introduction

Tsetse flies (*Glossina* spp.; Diptera: Glossinidae) are the cyclical vectors of two debilitating diseases in Africa, sleeping sickness in humans (human African trypanosomosis, HAT) and the cattle disease nagana (or African animal trypanosomosis, AAT) [1], [2]. Nagana, and in certain areas also sleeping sickness, has been a major obstacle to sub-Saharan African rural development and a severe constraint to agricultural production [3]. Due to the lack of effective vaccines and inexpensive drugs for HAT, and the development of resistance of the AAT parasites against available trypanocidal drugs [4], vector control remains the most efficient strategy for the sustainable management of these diseases [5].

Two attempts to establish of a colony of *Glossina pallidipes* (Austen) at the Insect Pest Control Laboratory (formerly Entomology Unit) of the Joint FAO/IAEA Programme of Nuclear Techniques in Food and Agriculture, Seibersdorf, Austria, were initially successful, but the colonies subsequently experienced a steady decline over several years and finally collapsed. Investigations revealed that up to 85% of both male and female flies showed symptoms of salivary gland hypertrophy (SGH+). This syndrome was first described in wild populations of *G. pallidipes*, but later detected in many tsetse species from different African countries [6]. The virus causing these symptoms was also associated with testicular degeneration and ovarian abnormalities [7]–[10] and affected the development, survival, fertility and fecundity of naturally or experimentally infected flies [6]. SGH+ *G. pallidipes* males were sterile and mostly unable to inseminate female flies. In *G. m. morsitans* and *G. m. centralis,* insemination by SGH+ males was also impaired [11]. In tsetse populations in nature, mother to offspring transmission, either trans-ovum or through infected milk glands, is thought to be the most likely mode of transmission of the virus [10], [12], [13], but in laboratory maintained flies horizontal transmission during membrane feeding is reported to be a significant route of virus infection [14], as each tray of blood is used to feed several successive sets of fly cages [15] leading to a build-up of virus in the blood diet.

The release of sterile males is a powerful and robust control tactic against tsetse flies but adequate competitiveness of the released sterile flies is a crucial prerequisite for success [16]. Loss in competitiveness can be compensated for by releasing higher numbers of sterile males per unit of surface, but this is not cost effective [17]. Although earlier studies report that the mating behaviour of SGH+ *G. m. morsitans* appeared to be normal in terms of mating duration and time to reach the jerking phase before separation [11], no information has been available on the mating propensity and competitiveness of SGH+ male *G. pallidipes*.

Multiple mating by wild female flies is another potential factor that may affect the efficacy of the SIT. However, it is significant only if the frequency of remating differs after a fertile or sterile mating, if females are able to discriminate between sterile and fertile males at the second mating, or if the females are able to use sperm selectively from the different matings. Information in the literature on the mating behaviour of tsetse flies in nature is scarce and the general assumption that wild female tsetse flies mate only once [18] has also received very little experimental attention.

In this paper the hypothesis that SGH in male *G. pallidipes* has a negative impact on their mating vigour or propensity to mating was examined. In addition, the multiple mating frequencies of female *G. pallidipes* were assessed in relation to SGH status of the male mates. All experiments were carried out in field cages to mimic the natural environment as closely as possible.

## Materials and Methods

### Tsetse flies

All experiments were carried out with flies from a *G. pallidipes* colony originating from pupae collected in Tororo, Uganda in 1975 and maintained since 1987 at the Insect Pest Control Laboratory, Seibersdorf, Austria. The adults were fed on warm, defibrinated, bovine blood (SVAMAN spol s.r.o., Myjava, 90701, SLOVAKIA) for 10–15 min three times per week using an *in vitro* membrane feeding system. The deposited larvae pupated and were incubated at 24°C until emergence [14], [19].

### Screening males of *G. pallidipes* for salivary gland hypertrophy symptoms

Although in female *G. pallidipes* external signs of SGH are not reliably visible, SGH can usually be detected by the swollen and pale appearance of the abdomen in male *G. pallidipes* that are older than 10 days and that have been starved for two days [20]. Males of *G. pallidipes* were collected and fed for 10–13 days post emergence, starved for two days then chilled at 4°C for 5–10 min, prescreened visually as previously described [20] and separated into two groups of flies: one with a high prevalence and one with a low prevalence of SGH. The two groups of males were kept at 24°C and 75% RH until used in mating tests. After mating, males observed to copulate were collected and dissected to confirm the presence of salivary gland hypertrophy. The absence of hypertrophy symptoms does not mean the absence of viral infection in the flies as many individuals are infected but asymptomatic [21].

### Mating and remating experiments

Mating experiments were conducted in cylindrical netted field cages, 2.9 m in diameter and 2.0 m high, placed in a laboratory rearing room at 24°C and 60–65% RH and containing a potted citrus plant to provide resting sites. All mating experiments were carried out between 9.30 am and 12.00 noon as previously described [22].

**Figure 1:**
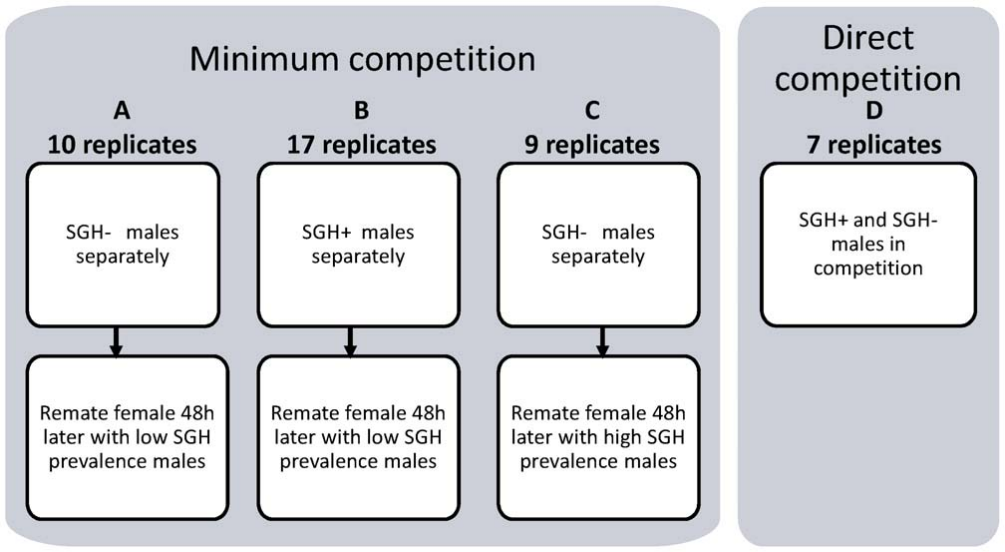
Schematic representation of the design of experiments A, B and C (minimum competition) and D (direct competition).

In each experiment, 7–day old virgin females were released in the field cage with 15–day old males from one of the groups described above at a ratio of 1 female: 1 male and 48 hours later given the opportunity to mate again. The salivary gland status of the males was determined by dissection after the initial mating. Only the female flies that had mated with SGH+ males from the high prevalence group and females that had mated with SGH− males from the low prevalence group were retained for remating; the remaining females (mated with SGH+ in the low prevalence group and with SGH− in the high prevalence group) were dissected to determine the spermathecal fill status. The remating experiments used a similar protocol as described above but at a ratio of 1 female: 2 males. The mating period was recorded and male dissections were carried out as described above. For the remating tests, three mating combinations were tested: (A) females that had first mated with SGH− males were given the opportunity to remate with low SGH prevalence males (B) females that had first mated with SGH+ males were given the opportunity to remate with males from the low SGH prevalence group, and (C) females that had first mated with SGH− males were given the opportunity to remate with males from the high SGH prevalence group (**Figure 1**).

Hypertrophied males cannot be distinguished visually with complete reliability and accurate identification of individual males as being either SGH+ or SGH− required destructive dissection and observation of salivary glands. As a consequence the high prevalence (SGH+ males) group contained a number of SGH− males and the low prevalence group contained some SGH+ males. There were differences in the performance of these males in the first experiments (without competition), so in order to compare the relative competitiveness directly a second experiment with direct competition was conducted. High and low prevalence groups of 15 day-old males were released in equal numbers (30 flies of each group with one group marked with a dot of acrylic paint on the thorax) with 7 day-old virgin females to give a ratio of 1 female: 2 males in the field cage. The flies were observed for two and half hours and copulating pairs were collected in individual tubes, each tube being numbered to identify the individual male and female flies. Premating time and mating duration were recorded and after separation of the couples male flies were removed and dissected to determine salivary gland status.

The number of females that mated as a proportion of the total females in each replicate is an indication of the tendency of the flies to mate (proportion mating, PM). The relative mating index (RMI) was defined as the number of mating pairs accounted for by the salivary gland status category as a proportion of the total number of mating pairs. This measure represents the competitiveness of SGH+ males relative to the SGH− males and is equivalent to the Relative Sterility Index [22].

### Pre-mating time and mating duration

The period between the flies' release in the mating cage until copulation was recorded as pre-mating time. Once genitalia were engaged and the pair was *in copula*, the pair was collected into individual tubes. The type of male (low or high SGH prevalence), and the starting and separation time of each successful mating were recorded to the nearest minute. The mating duration was then calculated as the difference in minutes between ending and starting times. Once copulation ended, the male was removed and dissected for salivary gland status and the female was kept in the fly holding room for the remating experiment or dissection for salivary gland status and scoring of spermathecal contents.

### Insemination

The female flies were dissected in physiological saline solution under a binocular microscope and the insemination rate and spermathecal fill determined [23]. If there were any doubts when using the binocular microscope the spermathecae were removed and mounted on a microscope slide and viewed under 100× magnification. The spermathecal fill was scored to the nearest quarter for each spermatheca separately as empty (0), partially-full (0.25, 0.50 or 0.75) or full (1.0) and the quantity of sperm transferred was then computed as the sum of the two spermathecae scores.

### Environmental conditions in the field cage studies

The temperature was maintained at 24±0.5°C and the relative humidity ranged from 60 to 65% during the observation periods. Light intensity (provided by high-frequency fluorescent tubes) varied from 290 to 550 lux with areas under the PVC supporting frame and tree leaves recording lower light intensity.

### Data analysis

The number of matings achieved and spermathecal fill categories were tested for equality of performance by SGH+ and SGH− males using the goodness of fit statistic G [24]. Premating times and mating duration were normalized by a square root transform following Box-Cox analysis [25]. Transformed values were analyzed by ANOVA and means compared by t-test [25]. Tabulated values are presented as detransformed mean values with 95% confidence intervals.

## Results

### Screening of *G. pallidipes* males for SGH+

The prevalence of SGH+ in the *G. pallidipes* tsetse colony established in the Insect Pest Control Laboratory was reported previously at 4–10% [20], [21]. The results show that the male flies screened as SGH+ by external observation (high prevalence group) had 83% SGH+ prevalence when confirmed by dissection whereas the male flies screened as SGH− (low prevalence group) had 7% SGH+.

### Impact of SGH+ on *G. pallidipes* mating behaviour

#### a) Impact of SGH+ on male mating efficiency

**Figure 2:**
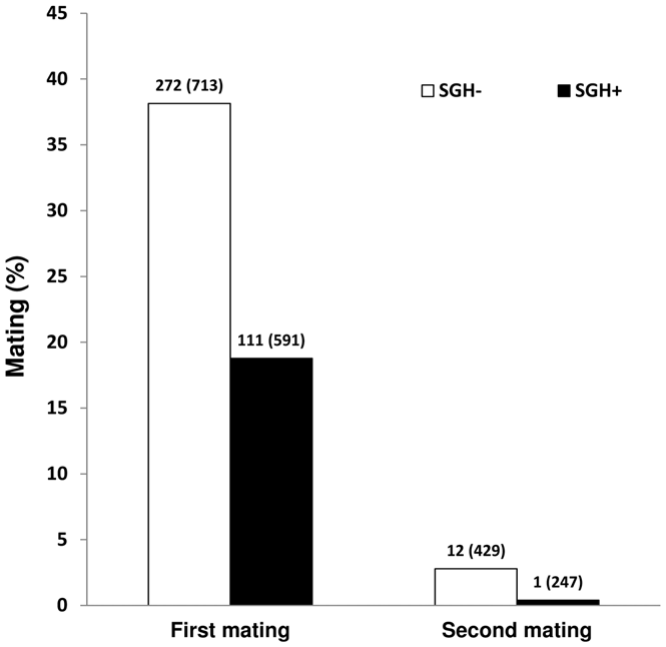
Mating percentage of SGH− and SGH+ males in the first and second matings. Numbers above columns indicate number mating and total number of males in category.

SGH+ male *G. pallidipes* were significantly less efficient in mating with 7 day-old females than SGH− male flies when released in a field cage either with a majority of males with SGH− (G = 10.77; d.f. = 1; *P* = 0.00103) or with a majority of males with SGH+ (G = 8.91; d.f. = 1; *P* = 0.002836) at a male: female ratio of 1∶1. Combined data of the no competition experiments indicate that only 19% of males of the high prevalence group mated, whereas 38% of the males of the low prevalence group succeeded in mating under field cage condition when they were offered the opportunity in a field cage (**Figure 2**). Males that managed to copulate had an SGH+ prevalence of 29% whereas the males that did not copulate showed an SGH+ prevalence of 52%, a highly significant difference from the mean rate of hypertrophy in these males (45.3%, G = 42.98; d.f. = 1; *P*≪0.001). No difference was observed in the SGH+ prevalence between mated and non-mated females.

Although the mating and remating experiments were designed to avoid competition between the two types of males, the released flies were actually a mixture of SGH+ and SGH− males (i.e. some males were classed as SGH− before the experiment started but proved to be SGH+ after dissection and vice versa, (**Table 1**). Whilst SGH+ males constituted at least 9% of the males in the low prevalence group, only 3.6% of matings were with these SGH+ males in the initial mating and none in the remating. Conversely, SGH− males constituted 13.7% and 30% of the males in the high prevalence group in the first mating and remating respectively but constituted over 23% and 87% of the mating pairs.

**Table 1:** Mating frequency by SGH status in field cage mating experiments.

SGH+ Prevalence	Reps	No of flies			Mating frequency		
		Females	Males	SGH+ ♂	No of pairs	SGH+ ♂	SGH− ♂
First mating							
Low	20	35.0±5.16	34.0±5.0	3.05±2.31	12.50±5.63	0.45±0.83	12.05±5.46**
High	18	34.44±5.11	34.11±5.00	29.44±4.40	7.39±3.52	5.67±2.57	1.72±2.14**
Second mating (after 48 hrs)							
A	10	9.10±3.73	17.00±9.29	2.00±2.45	0.10±0.32	0.00	0.10±0.32
B	18	5.44±2.53	11.67±5.31	1.11±1.32	0.50±1.47	0.00	0.50±1.47
C	10	14.40±5.08	29.50±9.30	20.60±10.20	0.80±1.32	0.10±0.32	0.70±1.06

**A:** Females mated with SGH− males and remated with low SGH prevelance group, **B:** Females mated with SGH+ males and remated with low SGH prevelance group, and **C:** Females mated with SGH-males and remated with high SGH prevelance group. Data are presented as mean values ± standard deviation.

** mating frequency differs between SGH+ and SGH− males in the same row at the 1% level. See Results section for full statistical analysis.

#### b) Impact of SGH+ on male competitiveness

In the direct competition experiment where SGH+ and SGH− males competed in almost equal numbers (a total of 192 SGH+ and 228 SGH− divided between 7 replicates), male flies with SGH+ were significantly less efficient in securing a mate (RMI of SGH+ = 0.204±0.053) in comparison to SGH− males (G = 30.6; d.f. = 6; *P*<0.0001). SGH+ males were substantially less competitive than SGH− males in both the low and high prevalence groups.

#### c) Impact of SGH+ on female mating efficiency

In general, the female flies in the no competition experiment that accepted a mate had a lower SGH+ prevalence (11.0%) than those that did not accept a mate (14.7%) although the difference was not significant (G = 1.817; d.f. = 1; *P* = 0.1776).

#### d) Impact of SGH on female remating behaviour

**Figure 3:**
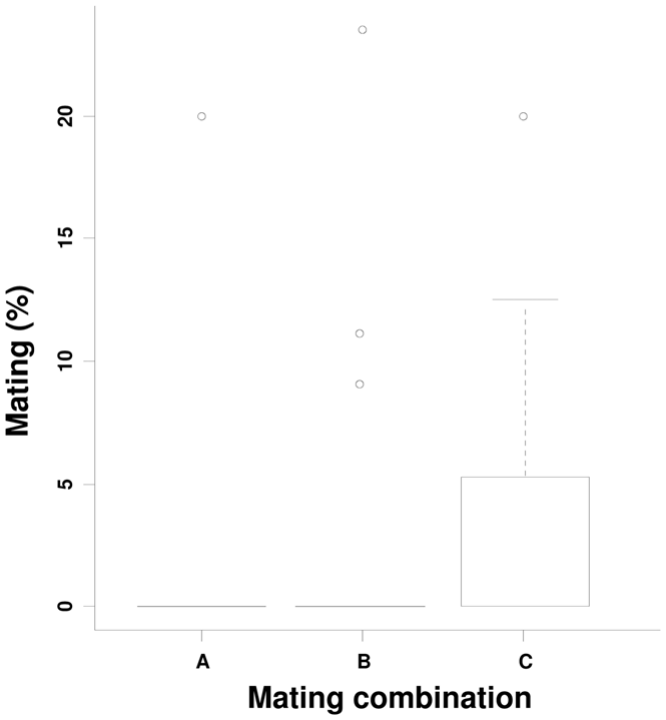
Impact of first and second mating combinations on the mating percentage in the second mating of *G. pallidipes*. A, Females mated with SGH− males and remated with SGH− males, B, Females mated with SGH+ males and remated with SGH− males, and C, Females mated with SGH− males and remated with SGH+ males.

The SGH status of the male from the first mating did not significantly influence the propensity of the females to remate when offered a second mating opportunity with 7-day old males in the field cage 48 hours after the first mating (SGH+ 2.7%, SGH− 2.0%, ns). However, for the very few females that did remate, there was a significantly greater number of SGH− than SGH+ males in the second mating (G = 5.701; d.f. = 1; *P* = 0.0169). These data indicate a high level of remating refractoriness in female *G. pallidipes* independent of SGH status of the male in the first mating (**Figure 3**).

### Pre-mating period and mating duration

Formation of mating pairs occurred soon after the release of males until the end of the observation period. The premating period and mating duration for the three mating combinations are shown in **Table 2**. As the pre-mating period and mating duration for SGH− and SGH+ males did not differ significantly in the experiments with no competition (F = 2.36; d.f. = 1,155; *P* = 0.126) values for all SGH− males were pooled and likewise for all SGH+ males. The pooled premating period for SGH− males was 49 and 64 minutes for first and second matings, and 64 minutes for SGH+ males.

**Table 2:** The mating duration and period before mating in minutes for SGH+ and SGH− male *G. pallidipes* in a field cage.

SGH+ Prevalence	Salivary gland status confirmed by dissection					
	Males with SGH−			Males with SGH+		
	*n*	Premating period	Mating duration	*n*	Premating period	Mating duration
	First mating					
Low	223	43.9–**48.3**–53.0a	27.5–**28.3**–29.3a	6	39.7–**57.5**–78.5a	25.9–**30.7**–36.0a
High	30	44.5–**55.7**–68.1a	22.1–**25.7**–29.4a	103	57.2–**64.3**–71.8a	25.0–**26.6**–28.2a
	Second mating (after 48 hrs)					
A	1	**74**	**27**	0		
B	3	22.7–**53.5**–97.2a	11.4–**23.8**–40.7a	0		
C	7	32.4–66**.2**–112a	27.1–**32.6**–38.6a	1	**3**	**29**

**A:** Females mated with SGH− males and remated with low SGH prevelance group, **B:** Females mated with SGH+ males and remated with low SGH prevelance group, and **C:** Females mated with SGH-males and remated with high SGH prevelance group.

Data are presented as detransformed mean values (in bold) and 95% confidence interval.

Mean values in the same column followed by the same letter do not differ at the 5% level. See Results section for full statistical analysis.

Pooled SGH− males had mean mating duration of 28 and 30 minutes in the first and second matings while SGH+ males had mean mating duration of 27 minutes. Only one SGH+ male copulated with a female mating for the second time with a premating period of 3 minutes and duration of 29 minutes. None of the differences was significant.

### Insemination efficiency

**Figure 4:**
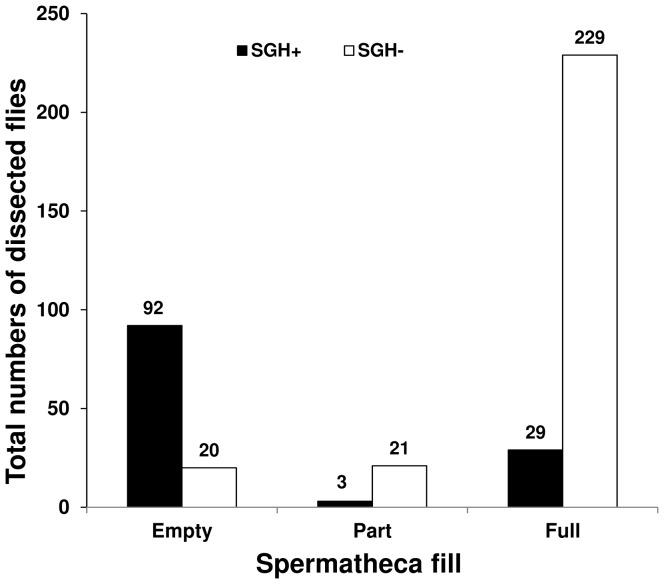
Impact of male SGH status on insemination rate (spermathecal fill) in *G. pallidipes* mated females.

The insemination efficiency, measured by the spermathecal fill in the first mating, was affected significantly by the male's salivary gland status (G = 139.8; d.f. = 2, *P*≪0.0001). The percentage of females with full spermathecae was significantly lower in females mated with SGH+ males (∼10%) than those mated with SGH− males (>80%) whereas, the percentage of females with empty spermathecae was higher in the females mated with SGH+ males (>80%) than those mated with SGH− males (∼10%) (**Figure 4**).

## Discussion

The SIT can only be successful if the released male insects are competitive with their wild counterparts and are able to locate virgin females to copulate. Tsetse flies, at least in *G. morsitans*, display a resource-based defence mating system, where the potential for mate monopolization by males is high due to the clumped distribution of the females and the resources (host animals) that are attractive to the receptive females [26]. Male mating success is largely determined by intra-sexual competition at these resources required by the females, both to intercept females and to prevent other males from gaining access to females [27]. Therefore, any reduction in male tsetse mating vigour will reduce the efficiency of the SIT component in integrated pest management programmes.

The data from our field cage studies, set-up to better mimic the natural environment as compared to small laboratory cages, indicates that the mating propensity and mating success of male *G. pallidipes* with SGH+ was reduced significantly as compared to SGH− male flies. Little more than half of males with SGH+ managed to secure a female mate as compared to SGH− males. These results are in agreement with previously reported data on mating in small cages in laboratory experiments [28], [29]. In addition, the results of our mating studies indicated that SGH+ males were less competitive as compared to SGH− males. This implies that releasing SGH+ *G. pallidipes* males would significantly decrease the effectiveness of a sterile male release programme or would increase its costs as the number of males needed would be almost double to get the same effect as compared to a programme that released SGH− males. Our observations are different from those obtained by Odindo [30] who observed no significant differences in mating performance between SGH+ and SGH− *G. pallidipes.* Our data also refute the speculation of Jura and Davies-Cole [11] that male flies sterilized by SGHV infection retain a competitive mating efficiency and may be useful in a sterile male release program. Our data and these of Odindo [30] and Jura and Davies-Cole [11] are most likely different because their experiments were conducted in small laboratory cages rather than field cages. The reduction in mating propensity of males with SGH+ is in agreement with the previous reported results that males of *H. zea* infected with the Hz-V2 virus are slower to approach healthy females and to attempt to mate compared with healthy males [31]–[33]. The reduction in mating propensity might be a result of reduction in the physical male activity i.e. flying and searching for females or negative selection by females against infected males. Odindo (1982) caught flies in the field with SGH+ that were fully engorged with blood, suggesting to him that flies with SGH+ showed normal flight and feeding behaviour under field condition [34]. However, there is no accurate data on the impact of virus infection on male flight performance. Moreover, as no data are available on the effect of virus infection on tsetse fly females' selection of males, further studies are required to understand the mechanism by which the virus infection reduces the mating propensity. Our tests were carried out with un-irradiated males, so further studies will also be needed to assess whether the addition of radiation treatment will further reduce the mating potential of SGH+ male *G. pallidipes* compared to SGH− males.

In addition to the lower mating propensity and success, the insemination rate was very low in females mated with SGH+ males, with more than 80% of the spermathecae empty. In comparison, more than 80% of the spermathecae of females mated with SGH− males were completely filled with sperm. These results confirm earlier data that the insemination potential of SGH+ *G. pallidipes* males is reduced drastically and males often were unable to inseminate females during mating, explaining the difficulties in maintaining adequate production levels in mass-reared colonies of this species [15]. The failure of males with SGH+ to inseminate females might be due to the lack of complete, functional spermatophores needed for sperm transfer as a result of short and small male accessory glands affected by the virus [9].

In a release programme, a high proportion of wild females that have mated with an SGH+ male will not have been inseminated and might have a higher inclination to remate, this time potentially with a fertile male. To test this hypothesis, the second part of the study looked at the remating potential of female *G. pallidipes* in relation to SGH status of their male mates. It was shown that female *G. pallidipes* had a very low remating frequency, irrespective of whether they had mated with a SGH− or a SGH+ male in the first mating. This accords with the results of Gillot & Langley (15) who showed in *G. morsitans* that inhibition of female remating was largely due to mechanical stimulation and was not dependent on the successful transfer of sperm or accessory gland factors but is in stark contrast to small cage observations of *G. pallidipes* [29], [35]. Jura & Davis-Cole [11] made similar observations with male *G. m. morsitans*, although those studies were done in small laboratory cages and not in an environment that allows mating choice. The low remating frequency of *G. pallidipes* females indicates again that in a sterile male release programme the first mating is very important and, hence, adequate competitiveness of the released sterile male flies is paramount.

The remating behaviour of female tsetse flies might be species-specific, as higher remating frequencies were obtained in similar remating experiments with *Glossina palpalis gambiensis* (GM, unpublished data). Dame and Ford [36], with chemically induced sterile sperm, demonstrated that multiple mating of female *G. morsitans* Westwood can occur under laboratory conditions, while males can copulate several times before depleting their sperm. Multiple mating was also reported in laboratory studies of *G. palpalis palpalis* Robineau-Desvoidy [37], [38] and for *G. austeni* [39], but refractory behaviour develops quickly in *G. morsitans* and it is unlikely that a female inseminated when seeking her first blood meal will permit mating to occur again when she seeks her second blood meal some days later [40]. Vreysen and Van der Vloedt [41] reported that in small laboratory cages, irradiated female *G. austeni* exhibited extensive multiple mating behaviour, and the ability of the irradiated flies for multiple mating decreases both with higher radiation dose (120 Gy) and treating flies later in life. For *G. pallidipes,* Jaenson [28] reported that it has been suggested that re-insemination of old females may take place in the field, but he mentioned that this hypothesis needs further elucidation. Recent work on *G. fuscipes fuscipes* has shown frequent polyandry in field samples [42].

SGH+ male *G. pallidipes* display a large swollen abdomen, that enables these males to be selected and separated visually [20]. A similar technique was used in our study to establish the two experimental groups with a high or low prevalence of SGH. The data indicates that the visual selection was quite efficient with 82% of males selected for the high prevalence group confirmed as being SGH+ at dissection. In the low prevalence group, only 6% of the dissected males were SGH+.

Although many tsetse species have been reported to be infected with SGHV and can display the SGH+ syndrome, only *G. pallidipes* has so far been shown to have a high prevalence of SGH+ in laboratory colonies, affecting colony growth and stability [6], [21]. This was epitomised by the collapse of two colonies of *G. pallidipes* in the Insect Pest Control Laboratory in 1987 and 2002 associated with a high prevalence of SGH syndrome The results presented in this paper clearly indicate that in addition to colony maintenance problems, releasing SGH+ *G. pallidipes* flies will also reduce the efficiency of a sterile male release programme and highlights again the need for an effective virus management strategy to maintain the viral load in colonies low enough that male flies can be released that are SGH−.

These studies present for the first time the impact of salivary gland hypertrophy virus infection on the mating behavior of *G. pallidipes* in field cages. The results support the following conclusion: (i) SGH+ males are less successful and less competitive in mating compared to SGH− males, (ii) SGH+ males did not inseminate the females when successful in mating and (iii) females mated with either SGH+ or SGH− males, with empty or full spermathecae, did not in general accept a second mating. These results also indicate the urgency of developing a virus management strategy not only to be able to effectively mass-rear *G. pallidipes* but to produce males with acceptable quality to be used in SIT release programmes.
